# Efficacy of partially hydrolyzed guar gum (PHGG) supplemented modified oral rehydration solution in the treatment of severely malnourished children with watery diarrhoea: a randomised double-blind controlled trial

**DOI:** 10.1186/s41043-015-0003-3

**Published:** 2015-05-01

**Authors:** Nur Haque Alam, Hasan Ashraf, Mohammad Kamruzzaman, Tahmeed Ahmed, Sufia Islam, Maryam Kadjar Olesen, Niklaus Gyr, Remy Meier

**Affiliations:** 1 International Centre for Diarrheal Disease Research Bangladesh (icddr,b), Dhaka, Bangladesh; 2 Department of Pharmacy, East West University, Dhaka, Bangladesh; 3 Nestle Health Care Nutrition, Gland, Switzerland; 4 Department of Internal Medicine, University Hospital, Basel, Switzerland; 5 Department of Gastroenterology, Hepatology and Nutrition, Kantonsspital, Liestal, Switzerland

**Keywords:** Diarrhoea, Severe malnutrition, Children, Partially hydrolyzed guar gum (PHGG)

## Abstract

**Objectives:**

To examine whether PHGG added ORS reduce duration of diarrhoea, stool output and enhance weight gain.

**Methods:**

In a double-blind controlled clinical trial, 126 malnourished children (weight for length/weight for age < −3 Z-score with or without pedal edema), aged 6 – 36 months with acute diarrhoea <7 days were studied in two treatment groups; 63 received modified WHO ORS (Na 75, K 40, Cl 87, citrate 7, glucose 90 mmol/L) with PHGG 15 g/L (study group); 63 received modified WHO ORS without PHGG (control). Other treatments were similar in both groups. The study protocol was approved by Ethics Committee of icddr,b; the study was carried out at the Dhaka Hospital.

**Results:**

The mean duration of diarrhoea (h) was significantly shorter in children of the study group (Study vs. control, mean ± SD, 57 ± 31 vs. 75 ± 39, p = 0.01). Although there was a trend in stool weight reduction in children receiving ORS with PHGG (study vs. control, stool weight (g), mean ± SD; 1^st^ 24 hour, 854.03 ± 532.15 vs. 949.11 ± 544.33, p = 0.32; 2^nd^ 24 hour, 579.84 ± 466.01 vs. 761.26 ± 631.64, p = 0.069; 3^rd^ 24 hour, 385.87 ± 454.09 vs. 495.73 ± 487.61, p = 0.196), especially in 2^nd^ 24 h period, the difference was not statistically significant. The mean time (day) to attain weight for length 80% of NCHS median without edema was significantly shorter in the study group (study vs. control, mean ± SD, 4.5 ± 2.6 vs. 5.7 ± 2.8, p = 0.027).

**Conclusion:**

PHGG added to ORS substantially reduced duration of diarrhoea. It also enhanced weight gain. Further studies might substantiate to establish its beneficial effect.

**Clinical trial registration number:**

NCT01821586

## Background

The median case fatality rate of severely malnourished children has remained as high as 20 to 26% for many years, despite improved understanding of the pathophysiology and treatment of these children [1]. It only highlights the widespread failure to use treatments that have been known for many years. However, there has been a decline in the case fatality rate with the use of standardized protocol for the treatment of severely malnourished children [2]. This standardized protocol prepared by World Health Organization [3] addressed overall management of severely malnourished children compiling the existing recommended treatment guidelines such as appropriate rehydration, infection control, appropriate feeding and micronutrient supplementation. But, there is a scope of further reducing the case fatality rate and enhance recovery from malnutrition by improving the treatment of common associated illnesses such as diarrhoea. Malnutrition predisposes to increased incidence and duration of illnesses, as well as to increased prevalence of prolonged illnesses [4]. Since diarrhoea is associated with further loss of weight and deficiency of macro- and micronutrients, early recovery along with lessening of diarrhoea severity will help to prevent further weight loss and deficiency of these nutrients in severely malnourished children. Additionally, severely malnourished children have depleted potassium stores, and diarrhoea further depletes the serum potassium concentration [5]. Early recovery from diarrhoea will prevent further deficits of serum potassium concentration and total body stores.

Current recommended ORS containing salts and glucose is effective in correcting dehydration even in severely malnourished children. Efforts have been made to further improve the efficacy of ORS in terms of reducing the duration of diarrhoea and stool output. One study [6] with WHO-ORS containing partially hydrolyzed guar gum (PHGG) and another [7] PHGG supplemented comminuted chicken diet have shown to reduce diarrhoea duration and stool output in children with acute and persistent diarrhoea, respectively. PHGG is a soluble fiber and its addition doesn’t alter the clarity of the solution, neither its taste. PHGG is produced by enzymatic hydrolysis of guar gum which is obtained from guar seed- one variety of legumes for use as a food additive and as a source of dietary fiber [8]. The added PHGG in the ORS or diet escapes digestion in the small intestine and enter into the colon where the fibers are expected to be fermented by colonic bacteria producing short chain fatty acids (SCFAs) [9]. SCFAs will stimulate sodium and water absorption in the colon [10-12] leading to early recovery from diarrhoea. In addition, SCFAs have trophic effect in the colonic mucosa, which utilize SCFAs as fuel thus enhancing nutritional recovery.

We hypothesized that the addition of PHGG to ORS will enhance recovery from diarrhoea and malnutrition of the severely malnourished children with acute diarrhoea. In this study we have used the current WHO/UNICEF recommended ORS formulation (Na^+^ 75) with increased concentration of potassium (K^+^ 40) for a better alternative in the management of severely malnourished children with watery diarrhoea in terms of correcting hypokalemia and preventing symptomatic hyponatraemia.

## Methods

### Study design

This was a randomized, double-blind, controlled clinical trial to assess the efficacy of (i) modified WHO ORS plus PHGG (Currently WHO recommended ORS with some modification, Na 75 mmol/L, Cl 87 mmol/L, K 40 mmol/L, citrate 7 mmol/L, Mg 3 mmol/L, Zn 300 μmol/L, Cu 45 μmol/L, glucose 90 mmol/L) plus PHGG 15 mmol/L - Study group) compared with ii) modified WHO ORS (Na 75 mmol/L, Cl 87 mmol/L, K 40 mmol/L, citrate 7 mmol/L, Mg 3 mmol/L, Zn 300 μmol/L, Cu 45 μmol/L, glucose 90 mmol/L without PHGG - control group) in the treatment of acute watery diarrhoea in children with severe malnutrition. The calculated osmolarity was 302 mosmol/L for both the solutions, because the PHGG does not change the osmolarity. The study was conducted at the Dhaka Hospital of the International Centre for Diarrhoeal Disease Research Bangladesh (icddr,b) from July 2007 to December 2009.

### Study population

Children of either sex, aged 6 months to 36 months, presenting with acute watery diarrhoea (defined as 3 or more watery stool for 24 hours) of <7 days duration, some or severe dehydration, weight for length < −3 Z-score/weight for age < −3 Z- score, were eligible for the study. The children with bloody diarrhoea, severe diseases (severe sepsis, meningitis, severe pneumonia with respiratory distress requiring intensive care and ancillary support such as O_2_ inhalation, oropharyngeal suction etc.) were excluded from the study. Written informed consent was obtained from parents/legal guardian of each of the participating children. The study protocol was approved by the Research Review Committee (RRC) and Ethical Review Committee (ERC) of icddr,b.

### Sample size

Based on the results of a clinical trial [13] of ORS solution in severely malnourished children with acute watery diarrhoea (the mean ± SD duration of diarrhoea was 66.4 ± 32.3 h), we anticipated a 25% reduction in diarrhoea duration in children receiving PHGG-added ORS. Considering 5% level of significance and 80% power and 5% drop out the sample size was estimated to be 63 in each group.

### Randomization

After eligibility was confirmed the patient was randomized to receive, either i) modified WHO-ORS plus PHGG (study group) or ii) modified WHO-ORS alone (control group). An experienced statistician at icddr,b, not involved in the study, prepared the randomization list using the randomization table. The name of intervention was indicated on a slip of paper, kept inside the sealed envelope. The sealed envelopes were supplied to the pharmacists of Dhaka Hospital, not involved in the study in any way, to prepare the solutions, which were identical in appearance and similar in taste. They labeled the bottles with the assigned random number, patient’s name, and hospital registration number and handed them over to the study staff for ready to drink. PHGG is a white powder completely soluble in water and does not make the change of color and taste of the solution at the concentration used in oral rehydration solution [6]. After 12 hours, unused ORS was measured and discarded and a fresh bottle of ORS was prepared for continuation of therapy, if required. Oral Rehydration Therapy (ORT) was continued until resolution of diarrhoea but up to a maximum of 7 days. The children with some dehydration was randomized immediately after admission and children with severe dehydration were given intravenous (IV) fluid immediately after admission and was randomized as soon as the child was out of hypovolaemic shock (improvement of alertness, able to drink and countable radial pulse) and the signs of severe dehydration has disappeared but not later than 4 hours. After 4 hours, if the children still have signs of severe dehydration were excluded from the study.

### Baseline information

Children fulfilling the eligibility criteria were admitted to the Research Ward of the Dhaka Hospital of icddr,b and stayed in the hospital throughout the study period until discharged. One of the investigators or research physician reconfirmed children’s eligibility for inclusion in the study. She/he took a detailed medical history of the enrolled children to determine the duration of and type of diarrhoea and its frequency; duration and frequency of vomiting; and presence of other symptoms such as fever, feeding difficulties, and treatment received for the illness before admission; and performed a thorough physical examination including assessment of dehydration according to the modified WHO guidelines (14) used in icddr,b and also the anthropometry performed and recorded.

### Laboratory tests

Laboratory tests included blood for determination of Hct%, total and differential WBC count, serum total protein, albumin and glucose, and serum electrolytes; stool for microscopy (including *Cryptosporidium* and *Giardia lamblia*) and culture for *Shigella*, *Salmonella*, *Vibrio cholerae*, and ELISA for rotavirus and urine microscopy were performed on admission. Serum electrolytes were repeated after 24 hours and 48 hours, if indicated after initiation of the ORS therapy. Other relevant investigations such as chest X-ray and blood culture were done, only if clinically indicated.

### Case management

We followed the standardized protocol for management of severely malnourished children used at icddr,b Dhaka Hospital [2,3]. Dehydration was assessed according to the modified WHO guidelines [14,15] followed in the hospital. In children with some dehydration, the fluid deficit was corrected with the assigned ORS in an amount 10 ml/kg/hour for the first two hours, then 5 ml/kg/hr until the deficit was corrected. In addition, ongoing stool losses were replaced with the assigned ORS 5–10 ml/kg after each watery stool. For high purging patient, the ORS intake was adjusted according to the on going stool loss. ORS therapy was continued until diarrhoea ceased. Children with severe dehydration were initially rehydrated with IV fluid (Cholera Saline containing Na 133 mmol/L, Cl 98 mmol/L, K 13 mmol/L, acetate equivalent to HCO3 48 mmol/L) until the patient was out of hypovolaemic shock (shock in these children was identified by altered consciousness/ lethargy, cold, clammy hands and feet, uncountable or absent radial pulse etc.) or disappearance of signs of severe dehydration. Then the rest of the rehydration was performed with ORS as for rehydration in patients with some dehydration, as described above. Children without apparent extra-intestinal infection received Injection ampicillin 100 mg/kg/24 h in 3 divided doses and Injection gentamicin 5 mg/kg/24 h in 2 divided doses for 5 days (as per protocol for management of children with severe malnutrition in icddrb and WHO). Children with lower respiratory tract infection received Injection Ceftriaxone 75 mg /kg/day for 5 days, and those with cholera initially diagnosed by positive Dark-field microscopy received syrup Azithromycin 20 mg/kg once within 20 minutes of randomization.

Mothers were advised to continue breastfeeding. Supplementary feeding with a locally prepared therapeutic diet of formula milk (cow’s milk, rice powder mixture, energy 70 kcal/100 ml) was given in an amount of 10 ml/kg for each feed two hourly for day 1 and increased slowly up to 150 Kcal/kg/day over 7 days according to demand. If the child had poor appetite, or was weak, or had painful stomatitis or glossitis, food was delivered through nasogastric tube until able to take food orally. Additionally, semisolid cooked foods (mixture of rice, lentil, vegetables etc.) were given to older children during convalescent and rehabilitation phase.

All children were given vitamins and minerals as per protocol [2,3]. Hypoglycemia (blood glucose <3.0 mmol/L) was managed with 50 ml 10% glucose orally or by nasogastric tube. Injection 25% glucose 2 ml/kg was given I/V, if the level of blood glucose was below 1.5 mmol/L. Hyponatraemia (serum sodium <115 mmol/L with or without symptoms) was managed with hypertonic saline such as Injection 3% NaCl at the rate of 12 ml/kg I/V slowly over 4 hours.

### Measurements

#### Fluids intakes (IV, ORS and water)

IV fluid was infused through a calibrated soluset, the amount infused was recorded every 6 hours if the patient was receiving I/V fluid; ORS was supplied after measuring with a calibrated cylinder and the amount of intake was noted every 6 hours, if any left over that was deducted from previous offer. Water intake was also measured in a similar way.

#### Output (stool, urine and vomitus)

Stool was collected in a bucket of known weight beneath the cholera cot with a central hole and measured every 6 hours with an electronic scale of a precision of 1 g. Urine was collected by pediatric urine collector (PUC bag) and measured with a calibrated cylinder in ml. Vomitus was collected in a pre-weighed bowl and measured with an electronic scale.

The children were offered a defined food of known calorie after measuring with an electronic scale of precision of 1 g. Any left over was measured and subtracted from the amount offered and amount ingested was recorded every 6 hours. Nude body weight was measured at admission, after rehydration and every 6 hours until recovery from diarrhoea and then at the end of every 24 hours and at discharge. Clinical evaluation was performed every morning and evening. Resolution of diarrhoea was defined as the passage of two consecutive soft/formed stool or no stool for 12 hours. Therapeutic success was defined as the cessation diarrhoea within 7 days of inclusion in the study treatment. Duration of diarrhoea was calculated in hours from the time of randomization to the last watery or loose stool within 7 days. Children were considered withdrawn from the study if their parents or legal guardian withdrew consent, or transferred to other units of the hospital for treatment of any complications; data (intakes and outputs) of such patients up to the time of withdrawal were included in the analysis (intent to treat analysis). Data of the children who failed to recover within seven days (study period) were also included in the analysis for a maximum of seven days; those were labeled as therapeutic failures and were treated in the hospital until recovery. All patients after cessation of diarrhoea and control of other associated infection were transferred to nutrition rehabilitation unit. The children were discharged from the hospital as soon as they attained weight for height measurement of 80% of NCHS median standard or ≥ −2 Z-score without edema and free from other medical illnesses. (This is also the existing criteria used for the severely malnourished children with diarrhoea attending the icddr,b, Dhaka Hospital). After discharge, they were also asked to attend our existing nutritional follow up unit (NFU) for further follow up. These follow ups were not recorded for analyses in this study.

### Clinical outcome

Main outcome measures were duration of diarrhoea, proportion of patient recovered within 72 hours, daily stool output and recovery from severe malnutrition (Attaining weight for length of 80% median or ≥ −2 Z-score without edema).

### Statistical methods

All data were entered into microcomputer and analyzed using the soft ware ‘statistical package for Social Science (SPSS PC+ version 10, Chicago, IL). Continuous variables were compared between groups with student’s *t*-test and nonparametric tests, categorical variables were compared by *χ*^2^ test. Kaplan-Meier survival curves were constructed for the duration of diarrhoea and compared with log rank test; a p value <0.05 was considered as significant.

## Results

### Recruitment and participant flow

**Figure 1:**
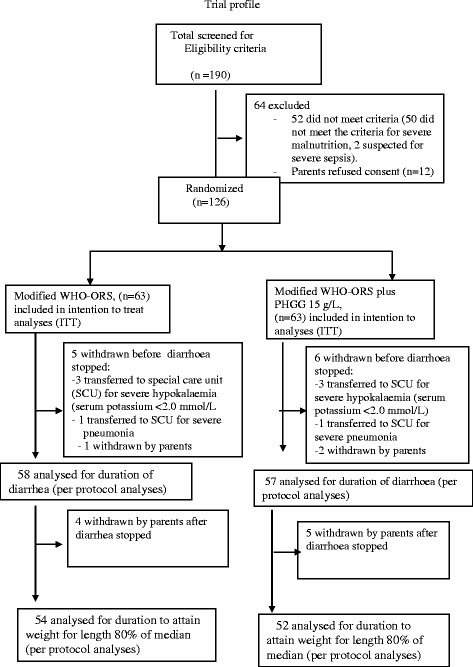
Trial profile.

In total 190 children attending the icddr,b Dhaka Hospital for treatment were screened for this study (Figure 1), of whom 126 children were randomized; 63 received modified WHO- ORS plus PHGG 15 g/L (study group) and 63 received modified WHO-ORS alone (control group). Reasons for non-randomization included failure to meet study inclusion criteria (n = 52; 50 did not meet the criteria of severe malnutrition and 2 suspected for severe sepsis) and refusal of parents consent (n = 12). During the study period after randomization, 6 patients were withdrawn from the study group before diarrhoea stopped. Of them 4 were transferred to special care unit (SCU) for management severe hypokalaemia (n = 3) and severe pneumonia (n = 1); withdrawn by parents (n = 2). In the control group, similarly 5 patients were withdrawn before diarrhoea stopped. Of them 4 were transferred to special care unit (SCU) for management severe hypokalaemia (n = 3) and severe pneumonia (n = 1); withdrawn by parents (n = 1). Therefore, 57 patients in the study group and 58 in the control group were included for analysis (per protocol analysis) for comparison of the duration of diarrhoea. During the whole study period before the patients attained 80% of the median weight for length without edema, 11 patients were withdrawn from the study in study group, of them 6 withdrawn before diarrhoea stopped (mentioned above) and 5 were withdrawn by parents after diarrhoea stopped. In the control group, 9 patients were withdrawn from the study before they attained 80% of median weight for length without edema, of them 5 withdrawn before diarrhoea stopped (mentioned above), and 4 withdrawn by parents after diarrhoea stopped. So, 52 patients in the study group and 54 in control group were included for analysis (per protocol analysis) for comparison of duration to attain 80% of the median weight for length without edema.

**Figure 2:**
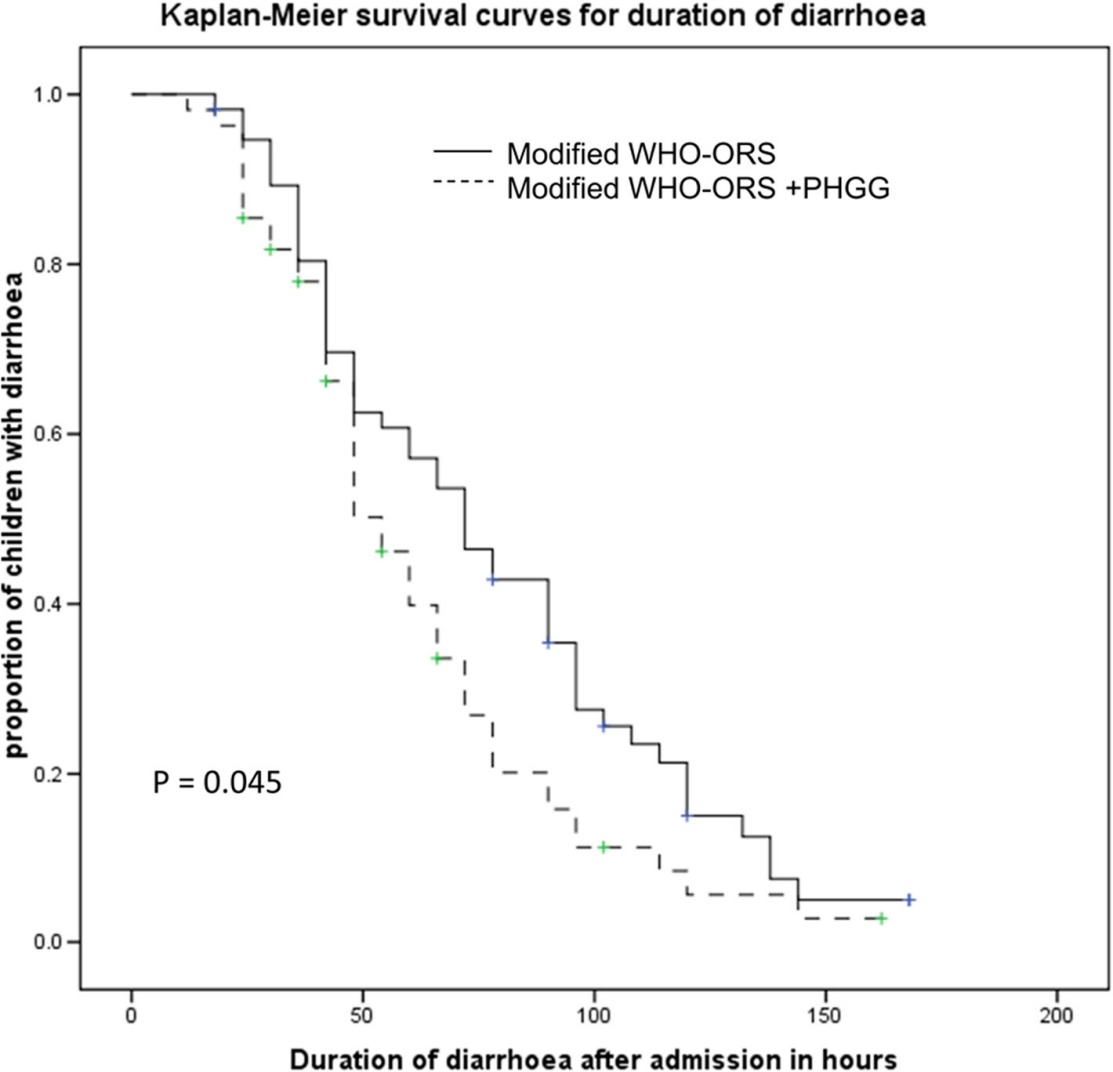
Kaplan Meier survival curves for duration of diarrhoea.

Baseline clinical characteristics such as age, body weight, nutritional status, duration of diarrhoea, dehydration status, breastfeeding status, and biochemical parameters, stool pathogen etc. were comparable between the treatment groups (Table 1). The mean duration of diarrhoea (h) was significantly shorter in children of the study group (p = 0.01) compared with children of the control group (Table 2). The proportion of children recovered from diarrhoea within 72 hours was more in the study group (p = 0.06), although the difference was not statistically significant (Table 2). The Kaplan –Meier Survival curve analysis for the duration of diarrhea also showed a similar trend towards reduced duration of diarrhoea (Figure 2) in children of the study group (p = 0.045, log rank test). The mean time (day) to attain weight for length 80% of NCHS median without edema was shorter in the study group (p = 0.027) compared to the control group (Table 2). There was a trend in stool reduction in children receiving ORS with PHGG, especially during the 2^nd^ 24 hour (p = 0.06), although the difference was not statistically significant (Table 3). The mean urine output (ml) was similar in the treatment groups (Table 3).

**Table 1:** **Admission characteristics of study patients (values are mean ± SD or numbers)**

**Variables**	**Modified WHO -ORS (n = 63)**	**Modified WHO-ORS + PHGG (n = 63)**
Age (month)	18. 3 ± 11. 26	17. 8 ± 10
Gender (male/female)	32/31	35/27
Admission Weight (kg)	6.0 ± 1.33	5.84 ± 1.13
Weight for age percent median	54. 9 ± 6.7	54.9 ± 5.38
Weight for length percent median	70.9 ± 5.3	70.8 ± 5.6
Weight for age Z score	−4.38 ± .68	−4.31 ± 0.63
Weight for length Z score	−3.14 ± 1.88	−2.76 ± 2.46
Presence of bi-pedal edema	46/63 (73%)	49/63(78%)
Serum Albumin (g/L)	39.7 ± 5.57	39.3 ± 6.12
Serum sodium (mmol/L)	140 ± 6.7	140 ± 5.8
Serum potassium (mmol/L)	3.54 ± 1.0	3.6 ± 1.0
Serum chloride (mmol/L)	105 ± 8.0	106 ± 7.0
SerumTCO_2_ (mmol/L)	16.4 ± 4.2	15.8 ± 3.6
Duration of diarrhoea before admission (hours)	39 **±** 18.2	40.0 **±** 15.68
Number of stool in last 24 hours.	14.36 **±** 6.00	14.02 **±** 6.09
Duration of vomiting before adm. (hours)	35.9 **±** 18.10	38.6 **±** 16.2
No. of vomit in last 24 hrs	7.7 **±** 4.1	7.4 **±** 3.4
Dehydration status on admission (Some/ Severe)	57/6	55/8
Breastfed prior to illness	43/63 (69%)	48/63(76%)
Stool pathogens		
*Vibrio cholera*	3/63	4/63
Rota virus	4/63	5/63
None identified*	56/63	54/63

*Stool tests to identify diarrhoeagenic *Escherichia coli* were not performed.

**Table 2:** C**omparison of outcome variables between two treatment groups**

**Variables**	**Modified WHO-ORS (n = 63)**	**Modified WHO-ORS + PHGG (n = 63)**	**Mean difference (95% CI)**	**p-value**
Duration of diarrhoea (hours) after admission	75 ± 39	57 ± 31	−18(−30, −4)	0.01
Number of children stopped diarrhoea within 72 hours	19/63(30%)	29/63(46%)	0.66(0.41 to 1.04)*	0.06
Time required to attain 80% of the median weight for length (days)	5.7 ± 2.8	4.5 ± 2.6	−1.2 (−2.27, −0.14)	0.027
Number of children with ORS failure/Requirement of unscheduled IV therapy	3/63 (5%)	4/63 (6.34%)	0.75(0.17 to 3.22)*	0.69

*Relative Risk (RR) (95% confidence interval).

Values are mean ± SD or numbers (%).

**Table 3:** **Comparison of output data (stool and urine) of the study patients**

**Variables**	**Modified WHO- ORS**	**Modified WHO- ORS + PHGG**	**Mean difference (95% CI)**	**P - Value**
Stool weight(g) 1^st^ 24 hour	949.11 ± 544.33	854.03 ± 532.15	−95.07(−284.90, 94.74)	0.32
Stool weight(g) 2^nd^ 24 hour	761.26 ± 631.64	579.84 ± 466.01	−181.42(−377.16, 14.30)	0.06
Stool weight (g) 3^rd^ 24 hour	495.73 ± 487.61	385.87 ± 454.09	−109.86(−277.30, 57.57)	0.19
Urine vol. (ml) 1^st^ 24 hour	237.75 ± 125.79	276.75 ± 189.11	38.99(−20.24, 98.23)	0.19
Urine vol. (ml) 2^nd^ 24 hour	395.17 ± 213.51	458.14 ± 246.25	62.96(−24.04, 149.98)	0.15
Urine vol. (ml) 3^rd^ 24 hour	397.50 ± 182.66	489.13 ± 304.20	91.63(−10.66, 193.92)	0.07

SD, standard deviation; CI, confidence interval.

Data are mean ± SD.

ORS intake was reduced in children of study group, however the differences were not statistically significant (Table 4). Other intakes (milk formula and water) were similar in both groups except increased mean water intake (ml) in the study group during the 3^rd^ 24 hour (p = 0.02) which was statistically significant (Table 4).

**Table 4:** **Comparison of intake data (ORS, milk formula and water) by study patients**

**Variables**	**Modified WHO- ORS**	**Modified WHO- ORS + PHGG**	**Mean difference (95% CI)**	**P – Value**
Ors intake (ml) 1^st^ 24 hour	825.95 ± 373.11	747.06 ± 388.30	−78.88(−213.17, 55.39)	0.24
Ors intake (ml) 2^nd^ 24 hour	609.84 ± 448.52	553.01 ± 446.84	−56.82(−214.70, 101.05)	0.47
Ors intake (ml) 3^rd^ 24 hour	396.98 ± 419.01	307.01 ± 372.11	−89.96(−230.35, 50.41)	0.20
Milk formula (ml) 1^st^ 24 hour	590.39 ± 111.78	597.45 ± 101.26	7.05(−32.36, 46.48)	0.72
Milk formula (ml) 2^nd^ 24 hour	609.01 ± 108.37	610.44 ± 141.86	1.42(−46.18, 49.03)	0.95
Milk formula(ml) 3^rd^ 24 hour	621.87 ± 108.90	619.23 ± 134.16	−2.63(−52.59, 47.32)	0.91
Water intake (ml) 1^st^ 24 hour	308.27 ± 122.16	344.21 ± 144.40	35.93(−13.44, 85.31)	0.15
Water intake (ml) 2^nd^ 24 hour	335.35 ± 120.30	330.09 ± 133.65	−5.26(−53.28, 42.75)	0.82
Water intake (ml) 3^rd^ 24 hour	315.72 ± 121.37	381.73 ± 154.52	66.00(9.22, 122.79)	0.02

SD, standard deviation; CI, confidence interval.

Data are mean ± SD.

## Discussion and conclusion

The study showed that PHGG when added to oral rehydration solution enhanced early recovery of acute diarrhoea in severely malnourished children in terms of reducing the duration of diarrhoea and stool output. Similar results were also reported when PHGG added ORS was used in the treatment of acute diarrhoea in non severely malnourished children [6], PHGG added comminuted chicken meat diet used in the treatment of persistent diarrhoea in children [7] and PHGG added ORS used in the treatment of adult cholera [16]. It has also been shown that PHGG added ORS in this study enhanced weight gain. So, the findings of the previous studies [6,7,16] and current study demonstrate that the beneficial effects of PHGG in the treatment of acute or persistent diarrhoea are consistent. It has also been shown beneficial effect of other unabsorbed carbohydrates (amylase resistant starch derived from maize) added ORS in the treatment of acute diarrhoea in children and adult cholera [17,18]. In Bangladesh, soluble fiber containing fruits (such as green plantain and semi ripe wood apple) are traditionally used as an anti-diarrhoeal food, with an expectation of beneficial effect in recovery. Recently one clinical study [19] in icddr,b examined the effect of cooked green banana (one variety used in Bangladesh as a vegetable) and pectin in the treatment of persistent diarrhoea in children. Both pectin and green banana have been shown to be beneficial in the treatment of persistent diarrhoea in children in terms of reducing the duration of diarrhoea and stool weight. Similar results have also been found in a community based trial, with green banana-supplemented diet in the home-management of acute (≤7 days duration) and prolonged (>7 days duration) diarrhoea in children [20].

The anti-diarrhoeal effect of soluble fiber/unabsorbed carbohydrate is thought to be mediated through their bacterial fermentation in colon, resulting in production of SCFAs. In the colon SCFAs facilitate absorption of sodium chloride and water [10,21], they also serve as an important source of energy for the colonocytes [22], and stimulate epithelial cell proliferation and exert a trophic effect on the colonic as well as small bowel mucosa [23,24].

The major limitation of the study was accuracy in collecting stool from female children. There might be some contamination of urine with stool. Careful recording of contamination was not possible. However, the distribution of female children in both the groups was similar in number due to the proper randomization effect that might take care for the comparability of the stool weight between the two groups. Other limitation of the study was that the stool test for diarrhoeagenic *Eschericia coli* was not performed and so majority of the unidentified pathogen in stool test might be diarrhoeagenic *Eschericia coli.* However, that should not influence the study results.

Despite the efficacy of ORS in correcting dehydration and preventing mortality that has already been established [25], its use has not yet achieved the widespread popularity, as it was initially expected [26,27]. Although this may be due to the lack of knowledge and appreciation of the effect of ORS, caregivers/parents in the community have been reluctant to use ORS as it does not substantially reduce diarrhoea. So, the present study was one of the efforts to find out an improved version of ORS that will reduce diarrhoea. To find out the best effective amylase resistant and fermentable carbohydrates for use in ORS, more clinical studies are to be carried out in future. Further more research on the issue of improved version of ORS should continue.

## References

[1] Schofield C, Ashworth A (1996). Why have mortality rates for severe malnutrition remained so high?. Bull World Health Organ.

[2] Ahmed T, Ali M, Ullah MM, Choudhury IA, Haque ME, Salam MA (1999). Mortality in severely malnourished children with diarrhoea and use of a standardized management protocol. Lancet.

[3] World Health Organization. Management of severe malnutrition: A manual for physicians and other senior health workers. Geneva; WHO. 1999, p.60

[4] Guerrant RL, Schorling JB, McAullife, de Souza MA (1992). Diarrhoea as a cause and effect of malnutrition: diarrhoea prevents catch up growth and malnutrition increases diarrhoea frequency and duration. Am J Trop Med Hyg.

[5] Zaman K, Islam MR, Baqui AH, yunus M (1985). Hypokalaemia in children with diarrhoea in rural Bangladesh. In J Med Res.

[6] Alam NH, Meier R, Schneider H, Sarker SA, Bardhan PK, Mahalanabis D (2000). Partially hydrolysed guar gum supplemented oral rehydration solution in the treatment of acute diarrhoea in children. J Ped Gatroentrol Nutr.

[7] Alam NH, Meier R, Sarker SA, Bardhan PK, Schneider H, Gyr N (2005). Partially hydrolysed guar gum supplemented comminuted chicken diet in the treatment of persistent diarrhoea: a randomised controlled trial. Arch Dis Child.

[8] Slavin JL, Greenberg NA (2003). Partially hydrolyzed guar gum: clinical Nutrition uses. Nutrition.

[9] Velazquez M, Davies C, Marett R, Slavin JL, Feirtag JM (2000). Effect of oligosaccharides and fibre substitutes on short chain fatty acids production by human faecal flora. Anaerobe.

[10] Roediger WE, Moore A (1981). Effect of short chain fatty acids on sodium absorption in isolated human colon perfused through vascular bed. Dig Dis Sci.

[11] Hoverstad T (1986). Studies of short chain fatty acids absorption in man. Scand J Gastroenterol.

[12] Cummings JH, Macfarlane GT (2002). Gastrointestinal effects of prebiotics. Br J Nutr.

[13] Dutta P, Mitra U, Manna B, Niyogi SK, Roy K, Mondol C (2001). Double blind, randomized controlled clinical trial of hypo-osmolar oral rehydration salt solution in dehydrating acute diarrhoea in severely malnourished children. Arch Dis Child.

[14] Alam NH, Ashraf H (2003). Treatment of infectious diarrhea in children. Paediatr Drugs.

[15] World Health Organization (1995). The treatment of diarrhea - A manual for physicians and other senior health workers.

[16] Alam NH, Ashraf H, Sarker SA, Olesen M, Troup J, Salam MA (2008). Efficacy of partially hydrolyzed guar gum-added oral rehydration solution in the treatment of severe cholera in adults. Digestion.

[17] Raghupathy P, Ramakrishna BS, Oommen SP, Ahmed MS, Priyaa G, DZiura J (2006). Amylase-resistant starch as adjunct to oral rehydration therapy in children with diarrhea. J Pediatr Gastroenterol Nutr.

[18] Ramakrishna BS, Venkataraman S, Srinivasan P, Dash P, Young GP, Binder HJ (2000). Amylase reistantant starch plus oral rehydration solution for cholera. New Eng J Med.

[19] Rabbani GH, Teka T, Zaman B, Majid N, Khatun M, Fuchs G (2001). Clinical studies in persistent diarrhea: dietary management with green banana or pectin in Bangladeshi children. Gastroenterology.

[20] Rabbani GH, Larson CP, Islam R, Saha UR, Kabir A (2010). Green banana-supplemented diet in the home management of acute and prolonged diarrhea in children: a community-based trial in rural Bangladesh. Trop Med Int Health.

[21] Binder HJ, Mehta P (1989). Short-chain fatty acids stimulate active sodium and chloride absorption in vitro in the rat distal colon. Gastroenteroloy.

[22] Roediger WE (1982). Utilization of nutrient by isolated epithelial cells of the rat colon. Gastroenterology.

[23] Sakata T (1986). Effects of indigestible bulk and short chain fatty acids on the tissue weight and cell proliferation rate of the digestive tract in rats. J Nutr Sci Vitaminol.

[24] Roediger WE, Rae DA (1982). Trophic effect of short chain fatty acids on mucosal handling of ions by the defunctioned colon. Br J Surg.

[25] Victora CG, Bryce J, Fontaine O, Monasch R (2000). Reducing deaths from diarrhea through oral rehydration therapy. Bull World Health Organ.

[26] Howteerakul N, Higgin Botham N, Freeman S, Dibley MJ (2003). ORS is never enough: physician rationales for altering standard treatment guidelines when managing childhood diarrhea in Thailand. Soc Sci Med.

[27] Larson CP, Saha UR, Islam R, Roy N (2006). Childhood diarrhea management practices in Bangladesh: private sector dominance and continued inequities in care. Int J Epidemiol.

